# Chromosome Sequence of *Borrelia miyamotoi*, an Uncultivable Tick-Borne Agent of Human Infection

**DOI:** 10.1128/genomeA.00713-13

**Published:** 2013-09-12

**Authors:** Fong Hue, Arash Ghalyanchi Langeroudi, Alan G. Barbour

**Affiliations:** Departments of Microbiology and Molecular Genetics and Medicine, University of California Irvine, Irvine, California, USA

## Abstract

*Borrelia miyamotoi* is a newly recognized agent of human disease. *B. miyamotoi* strain LB-2001, an isolate from the tick *Ixodes scapularis*, was propagated in mice. The sequence of the chromosome was determined by next-generation sequencing of DNA isolated from whole blood. The sequence established that *B. miyamotoi* is a relapsing fever group species.

## GENOME ANNOUNCEMENT

The spirochete genus *Borrelia* comprises several species falling into one of two major clades (1). One clade contains Lyme disease (LD) agents, such as *Borrelia burgdorferi*, which are transmitted by ixodid ticks. The other major group contains species, such as *Borrelia hermsii*, that cause relapsing fever (RF) in humans and are transmitted by argasid ticks. This second clade also contains three species that exclusively use ixodid ticks as vectors: *Borrelia theileri*, *Borrelia lonestari*, and *Borrelia miyamotoi* (2, 3). The last species is transmitted by *Ixodes* species ticks, including *Ixodes scapularis*, the vector of LD in eastern North America (4). Recently, *B. miyamotoi* has been reported to be associated with symptomatic febrile illnesses in humans in Eurasia and North America (5–8). While genomes of five argasid tick-transmitted species have been reported, there has been little characterization of any ixodid tick-transmitted species in this clade.

*B. miyamotoi* strain LB-2001 is uncultivable at the present. To obtain cells for DNA, we infected severe-combined immunodeficient mice (C.BKa-*Igh*^*b*^/IcrCrl) as described (9). When bacterial densities in the blood reached ~10^7^ cells/ml, the mice were terminally exsanguinated under anesthesia. Total DNA was extracted from citrated whole blood with the Qiagen DNeasy blood and tissue kit (Valencia, CA) and then treated with RNase I. The library was produced with the Ion Xpress Plus fragment library kit with size selection by electrophoresis before emulsion PCR on an Ion OneTouch apparatus (Life Technologies, Carlsbad, CA). Sequencing was carried out on an Ion Torrent PGM apparatus with 200-bp nucleotide chemistry and four Ion 316 Chips (Life Technologies). The 9,872,663 trimmed reads of 50 to 260 nucleotides (nt) that were obtained were filtered with the 2.6-Gb *Mus musculus* sequence of Genome Reference Consortium Mouse Build 38 (assembly identification [ID] GCA_000001635.2), yielding 1,635,742 (17% of total) unmapped reads. With the Assembly Cell algorithm of the CLC Genomics Workbench v.6 (CLC bio, Denmark), these were assembled *de novo* into 14 chromosomal DNA contigs of average length 64,712 bp. Gaps between the contigs were filled by PCR amplification with custom primers and Sanger sequencing of products, as described (10). For 907 nonoverlapping windows of 1,000 bp, the median and mean coverage were 79×, with a standard deviation of 9. The prediction of protein-coding sequences (CDSs) and annotation were performed by the Prokaryotic Genome Automatic Annotation Pipeline with GeneMarkS + v.2.1 (http://www.ncbi.nlm.nih.gov/genome/annotation_prok), followed by manual annotation.

The linear chromosome sequence comprises 907,294 bp, with a G+C content of 28.7%. The chromosome contains 808 CDSs, 31 tRNAs, and 3 rRNAs that are in the same gene order as on the chromosomes of *B. hermsii* (accession no. CP000048) and other *Borrelia* spp. A major shift in the GC skew at ~455 kb was consistent with an origin of replication. The phylogenetic placement of *B. miyamotoi* within the clade of RF species rather than the LD species clade was confirmed by the finding of 15 CDSs that were unique to *B. hermsii* and only one CDS unique to *B. burgdorferi* (GenBank accession no. AE000783).

### Nucleotide sequence accession number.

The complete chromosome sequence of *B. miyamotoi* LB-2001 has been deposited in the GenBank/DDBJ/EMBL database under the accession no. CP006647.
